# Characteristics of pyogenic odontogenic infection in patients attending Mulago Hospital, Uganda: a cross-sectional study

**DOI:** 10.1186/s12866-015-0382-z

**Published:** 2015-02-25

**Authors:** Richard Kityamuwesi, Louis Muwaz, Arabat Kasangaki, Henry Kajumbula, Charles Mugisha Rwenyonyi

**Affiliations:** Department of Oral and Maxillofacial Surgery, Mulago Hospital, Kampala, Uganda; Department of Dentistry, School of Health Sciences, College of Health Sciences, Makerere University, Kampala, Uganda; Department of Microbiology, School of Biomedical Sciences, College of Health Sciences, Makerere University, P.O. Box 7072, Kampala, Uganda

**Keywords:** Antibiotics, Bacterial isolates, HIV, Odontogenic infection, Susceptibility

## Abstract

**Background:**

Predisposing factors of pyogenic odontogenic infection include dental caries, pericoronitis, periodontitis, trauma to the dentition and the supporting structures or complications of dental procedures. The infections are usually polymicrobial involving normal endogenous flora. We characterised pyogenic odontogenic infection in patients attending Mulago Hospital, Uganda.

**Results:**

Of the 130 patients, 62 (47.7%) were female. The most frequently involved fascial spaces were: the buccal, 52 (25.4%); submasseteric, 46 (22.4%) and the submandibular space, 36 (17.5%). Dental caries was the most prevalent predisposing factor, particularly of the lower third molar teeth. Viridans Streptococci Group and *Staphylococcus aureus* were the most frequent bacterial isolates: 23.5% and 19.4%, respectively. All Viridans Streptococci isolates were resistant to penicillin G, sulfamethoxazole/trimethoprim (cotrimoxazole), ampicillin and tetracycline, but susceptible to vancomycin. All *Staphylococcus aureus* strains were resistant to cotrimoxazole and ampicillin while retaining susceptibility to vancomycin, cefotaxime, linezolid, moxifloxacin and amoxicillin/clavulanate. Thirty five (26.9%) patients were HIV infected and the HIV status did not significantly influence the pattern of odontogenic infection.

**Conclusions:**

Dental caries was the most prevalent predisposing factor for pyogenic odontogenic infection. High prevalence of bacterial resistance to ampicillin and cotrimoxazole suggests the need for regular antibiotic susceptibility tests of isolates and rational use of antibiotics in the management of these infections. Prevention requires strengthening of oral health in the community.

## Background

Studies indicate that the majority of pyogenic oro-facial infections are due to odontogenic infection [1], which are usually due to dental caries, pericoronitis, periodontitis, trauma to the dentition and the supporting structures or complications from dental procedures [2]. Most such infections are usually polymicrobial in nature [1,3], although several studies indicate that the most common bacteria isolated in odontogenic infection are *Streptococcus, Peptostreptococcus, Eubacterium, Porphyromonus, Prevotella* and *Fusobacterium species* [4]; more recently, *Staphylococcus aureus* has also emerged as an oral pathogen [5].

The second and third permanent molars are the most commonly associated with pyogenic odontogenic infection [6]. Furthermore, the anatomic location of teeth involved may determine the fascial spaces to which the infection may spread [7]. Although a previous study [8] showed the submandibular space to be the most frequently involved with pyogenic odontogenic infection, other workers [9] have shown different fascial spaces to be more prevalent.

About 7 to 12% of antibiotic prescriptions in all infections are for odontogenic infection [10]. However, the prescriptions are usually empirical without routine culture and sensitivity tests. Regrettably, use of broad spectrum antibiotics has resulted in decreased sensitivity of oral bacteria with a growing number of resistant strains [11]. The development of resistance to antibiotics has a tremendous impact on the selection of antimicrobial agents for empirical therapy. In a bacterial susceptibility study, Phillips et al. [12] indicated distinct differences in resistance pattern related to individual hospitals, geographic regions and antibiotic prescribing regimen. The complications of pyogenic odontogenic infection such as suppurative mediastinitis, intracranial extension, cavernous sinus thrombosis, Lemierre syndrome, maxillary sinusitis, Ludwig’s angina, carotid artery erosion, osteomyelitis, retropharyngeal spread, airway obstruction, pleuropulmonary involvement, haematogenous dissemination with septic shock and disseminated intravascular coagulopathy indicate the potentially serious nature of these infections [13].

In Uganda, the few susceptibility studies done on oral bacterial isolates were from throat swabs [14] and whole saliva [15], but not specimens from pyogenic odontogenic infection. The World Health Organization prescription guidelines [16] and the Ministry of Health, Uganda clinical guidelines [17] recommend the use of phenoxymethylpenicillin, amoxicillin, erythromycin, metronidazole and procaine penicillin fortified in the management of dental abscesses, gingival infections, periodontitis and other perioral infections, but no studies have been done in Uganda to assess the efficacy of the antibiotics in pyogenic odontogenic infection. Additionally, it is reported that bacterial sepsis is common in immuno-deficiency [18] and already some studies [19] have investigated the relationship between human immuno-deficiency virus/acquired immuno-deficiency syndrome (HIV/AIDS) and pyogenic odontogenic infection elsewhere. The aim of the present study was to determine the microbiology of pyogenic odontogenic infection and perform antibiotic susceptibility tests in patients attending Mulago Hospital, Uganda.

## Methods

### Study design and setting

We studied 130 consecutively diagnosed in- and out-patients with pyogenic odontogenic infection attending the Oral and Maxillofacial Surgery Unit in Mulago Hospital from April 2011 to January 2012.

### Participants and procedures

Patients (n = 136) with age ranging from 1.5 to 89 years with pyogenic odontogenic infection were consecutively recruited into the study as they presented in the Oral and Maxillofacial Surgery Unit. The patients with immuno-suppressive conditions other than HIV/AIDS such as diabetes mellitus (n = 2), primary immuno-deficiencies (n = 1) and malignancies (n = 3) were excluded from the study leaving a sample of 130.

Demographic information was elicited from patients using a structured questionnaire. Evaluation of the socio-economic status of the patients was based on their occupation, education status and the average monthly income estimates as described in the Kuppuswamy’s Socioeconomic Status Scale [20]. All patients were examined for pyogenic odontogenic infection while lying supine in a dental chair by the principal investigator (RK). Patients were evaluated for general health status including cardiovascular and respiratory systems, vital signs, fever, dysphagia, dyspnea, odynophagia and malaise [7]. Measurement of inter-incisal opening to assess trismus was done using a pair of digital vernier calipers (Avery, United Kingdom). An opening of less than 20 mm was considered positive for trismus. A swelling was assessed by visual inspection and bimanual palpation. An abscess was confirmed through a combination of the following clinical features: a well circumscribed warm, tender swelling, fluctuant with a shiny overlying skin or erythematous mucous membrane and pus aspirate. Cellulitis was confirmed as a warm, hard, tender, diffuse swelling with a sero-sanguinous aspirate with flecks of pus. A fascial space involved with the infection was identified during clinical examination and intra-operatively as previously described [9,21]. An abscess was incised and drained through percutaneous incision after specimen collection. Peritonsilar abscesses (n = 5) were drained through incisions in the mucosa and carious teeth involved in the infection were extracted.

Ethical approval was obtained from Makerere University, School of Medicine Institutional Review Board and Mulago Hospital Ethics and Research Committee. Written consent was obtained from the adult patients and parents/guardians of children. They were also requested for voluntary counseling and testing for HIV. The nature and benefits of the study were explained to the patients in accordance with the Helsinki Declaration relating to the conduct of research on human subjects [22]. All the information collected was kept confidential and the patients had no personal identifiers.

### Laboratory methods

The bacterial isolation and susceptibility testing was performed at Makerere University College of Health Sciences Microbiology Laboratory and Mulago - Mbarara Teaching Hospitals’ Joint AIDS Program Laboratory.

All specimens were handled according to the Clinical Microbiology Laboratory Standard Operating Procedures [23]. The aspiration site was first cleaned with sterile gauze dipped in 70% ethyl alcohol solution and 4 ml of pus aspirated from the abscess into a disposable 10 ml syringe with a gauge 21 hypodermic needle (Puning Haiou Medical Appliance Co. Ltd, China). In case of cellulitis where no pus could be aspirated, sero-sanguinous fluid was augmented by injection of 3 ml of sterile normal saline into the swelling and aspirated. The specimens were delivered to the Laboratory, within 1 hr of collection for bacterial analysis. A portion of the specimen (3.5 ml) was immediately inoculated in a transport medium, Soybean casein digest broth - BD BACTEC™ Plus + Anaerobic/F Medium (Becton Dickinson and Co., Ireland). The remaining 0.5 ml of the specimen was retained in the syringe which was securely capped and used for Gram staining and Ziehl–Neelsen staining.

The specimen from the Soybean casein digest broth was inoculated onto 3 primary plates: 1) Sheep blood agar (Biolab, Hungary) and incubated in 5% CO_2_ at 37°C under aerobic conditions for 18 to 24 hr for isolation of aerobes, 2) MacConkey Agar (Biolab, Hungary) and incubated in 5% CO_2_ at 37°C under aerobic conditions for 18 to 24 hr for isolation and differentiation of Gram-negative enteric bacilli, and 3) Anaerobic agar (Biolab, Hungary) with 5% sheep blood and incubated at 37°C for 48 to 72 hr. The anaerobic conditions were generated by the BD GasPak™ EZ Anaerobe Container System (Becton Dickinson and co., USA).

Anaerobic bacteria were further cultivated on Anaerobic agar (Biolab, Hungary) with addition of 50 mg/ml kanamycin to selectively inhibit facultative anaerobes and aerobes while permitting growth of strict anaerobes.

Antibiotic sensitivity tests for the aerobic/facultative isolates were performed by the disk diffusion method of Kirby-Bauer [24] on Mueller Hinton agar (Biolab, Hungary). The interpretive categories were susceptible, intermediate and resistant depending on the growth inhibition zone diameters. Quality Control was based on American Type Culture Collection (ATCC) strains, namely, *E. coli* (ATCC 25922), *E.coli* (ATCC 35218), *S. aureus* (ATCC 25923), *P. aeruginosa* (ATCC 27853), *K. pneumoniae* (ATCC 700603), *S. pneumoniae* (ATCC 49619), *H. influenza* (ATCC 49247) and *H. influenza* (ATCC 49766).

### HIV testing and CD4+ T lymphocyte cell count

A 3 ml blood sample from each patient was drawn aseptically by venipuncture using disposable 5 ml syringe and gauge 23 hypodermic needle (Puning Haiou Medical Appliance Co. Ltd, China). Serological tests for HIV were performed using a standard HIV testing algorithm of two enzyme linked immunosorbent assays and confirmed with Western Blot.

The CD4+ T lymphocyte cell count was determined by flow cytometric analysis of blood samples using the BD MultiTEST CD3/CD8/CD45/CD4, a 4 color direct immunofluorescence reagent and TruCount tubes on a FACSCalibur™ using the manufacturer’s instructions.

### Statistical analysis

The independent variables were age, sex, place of residence, level of education, occupation, socioeconomic status, clinical signs and symptoms, site of origin of infection, HIV sero-status, predisposing factors, bacterial growth on culture, bacterial isolates, nature of infection, CD4+ T lymphocyte cell counts, bacterial sensitivity pattern, prior antibiotic therapy, possession of a prescription, fascial spaces involved and hospital admission status. The outcome variable included complications due to infection [7,25].

Data were analysed using Statistical Package for Social Sciences Inc. (SPSS version 19 for windows, Chicago, Illinois, USA). The independent sample *t* test and the Chi square statistics were used to assess any significant association based on quantitative variables. Multivariate logistic regression analyses were used to estimate the risk of independent variables of patients in developing complications due to pyogenic odontogenic infection. The level of significance was set at two sided 5%.

## Results

### Demographic characteristics

A total of 130 patients with pyogenic odontogenic infection were recruited into the study. The mean age of the patients was 29.5 (range, 1.5 to 89) years. The female patients constituted 47.7% (n = 62). Thirty-five (26.9%) of the patients including 12 women were HIV infected (Table 1). About 63.1% of the patients were in the lower socio-economic class and 60% either had no formal education or attained primary education (Table 1).

**Table 1:** **The frequency distribution of patients with pyogenic odontogenic infection according to demographic characteristics and other variables (n = 130)**

**Variable**	**Category**	**HIV + ve**	**HIV-ve**	**All**
		**n (%)**	**n (%)**	**n (%)**
Age group	≤35 years	24 (18.5)	71 (54.6)	95 (73.1)
	>35 years	11 (8.5)	24 (18.5)	35 (26.9)
Sex	Female	12 (9.2)	50 (38.5)	62 (47.7)
	Male	23 (17.7)	45 (34.6)	68 (52.3)
Level of education	Informal/Primary education	23 (17.7)	55 (42.3)	78 (60.0)
	Post primary education	12 (9.2)	40 (30.8)	52 (40.0)
Socio-economic status	Middle	13 (10.0)	35 (26.9)	48 (36.9)
	Lower	22 (16.9)	60 (46.2)	82 (63.1)
Predisposing factors	Dental caries	14 (10.8)	50 (38.5)	64 (49.2)
	Post extraction	8 (6.2)	23 (17.7)	31 (23.8)
	Trauma	1 (0.8)	7 (5.4)	8 (6.2)
	Periodontitis	3 (2.3)	3 (2.3)	6 (4.6)
	Pericoronitis	1 (0.8)	5 (3.8)	6 (4.6)
	Non specific	8 (6.2)	7 (5.4)	15 (11.5)
Prior antibiotic use	Yes	31 (23.8)	71 (54.6)	102 (78.5)
	No	4 (3.1)	24 (18.5)	28 (21.5)
Prescription^a^	Yes	21 (20.6)	28 (27.5)	49 (48.0)
	No	10 (9.8)	43 (42.2)	53 (52.0)
Hospital admission	No	26 (20.0)	80 (61.5)	106 (81.5)
	Yes	9 (6.9)	15 (11.5)	24 (18.5)
Bacterial culture	Positive	19 (14.6)	60 (46.2)	79 (60.8)
	Negative	16 (12.3)	35 (26.9)	51 (39.2)
Nature of infection^b^	Polymicrobial	3 (3.8)	8 (10.1)	11 (13.9)
	Not polymicrobial	16 (20.3)	52 (65.8)	68 (86.1)
Site of origin of infection^c^	Maxilla	9 (7.6)	24 (20.3)	33 (28.0)
	Mandible	20 (16.9)	65 (55.1)	85 (72.0)
Complication of infection	Yes	8 (6.2)	16 (12.3)	24 (18.5)
	No	27 (20.8)	79 (60.8)	106 (81.5)

^a^n = 102, ^b^n = 79, n^c^ = 118.

### Predisposing factors for pyogenic odontogenic infection

All patients presented with pain and swelling; 47.7% had fever; 55.4%, trismus and 16.9% had dyspnea. Most patients (72.0%) had infections originating from the mandible (Table 1). About half (49.2%, n = 64) of the patients had dental caries as the predisposing factor. In permanent dentition, caries was most frequently recorded in the lower left and right molar teeth: 22 (34.4%) and 16 (25%), respectively. The lower right third molar (n = 10, 15.6%) and the lower left third molar (n = 9, 14.1%) were the teeth most frequently involved with caries. In children with deciduous teeth, the upper and the lower right molars were the most involved teeth: 4 (6.3%) and 3 (4.7%), respectively. Thirty one patients including 8 with HIV infection had pyogenic odontogenic infection as a post tooth extraction complication. HIV infection was not a significant risk factor of developing post tooth extraction infections (Odds Ratio, 0.93, 95% CI: 0.44 - 2.32, p = 0.872).

### Bacterial isolates and levels of immunity

Bacteria were isolated from samples obtained from 79 (60.8%) including 19 HIV infected patients (Table 1). We found 98 bacterial isolates: 67 (84.8%) had 1 isolate; 6 (7.6%), 2 isolates; 5 (6.3%), 3 isolates and 1 (1.3%) had 4 isolates. Viridans Streptococci Group (23.5%, n = 23) and *Staphylococcus aureus* (19.4%) were the most prevalent isolates (Table 2). Seventeen HIV infected patients (8/9: male/female) with positive cultures had CD4+ T lymphocyte cell counts ranging from 22 to 1077 (mean 542.8 ± 330.7) cells/mm^3^ of blood. The frequency distribution of bacterial isolates from HIV infected patients according to levels of immunity are depicted in Table 3. There was no specific association between the bacterial isolates and CD4+ T lymphocyte cell counts (p = 0.445).

**Table 2:** **The frequency distribution of bacterial isolates from pyogenic odontogenic infection in patients with positive cultures according to HIV sero-status and sex (n = 98)**

**BacteriaI isolates**	**HIV-ve n (%)**	**HIV + ve n (%)**	**Male n (%)**	**Female n (%)**	**All n (%)**
**Facultative anaerobes**					
Viridans Streptococci group	18 (24.7)	5 (20.0)	12 (25.0)	11 (22.0)	23 (23.5)
*Staphylococcus aureus*	15 (20.5)	4 (16.0)	8 (16.7)	11 (22.0)	19 (19.4)
*Escherichia coli*	9 (12.3)	1 (4.0)	6 (12.5)	4 (8.0)	10 (10.2)
*Haemophilus influenzae*	5 (6.8)	2 (8.0)	2 (4.2)	5 (10.0)	7 (7.1)
*Staphylococcus epidermidis*	2 (2.7)	2 (8.0)	2 (4.2)	2 (4.0)	4 (4.1)
*Klebsiella pneumoniae*	3 (4.1)	1 (4.0)	2 (4.2)	2 (4.0)	4 (4.1)
*Enterococcus species*	2 (2.7)	1 (4.0)	1 (2.1)	2 (4.0)	3 (3.1)
*Streptococcus pneumoniae*	1 (1.4)	2 (8.0)	1 (2.1)	2 (4.0)	3 (3.1)
*Streptococcus aggalactiae*	2 (2.7)		1 (2.1)	1 (2.0)	2 (2.0)
*Proteus mirabilis*	1 (1.4)	1 (4.0)		2 (4.0)	2 (2.0)
*Streptococcus pyogenes*	1 (1.4)		1 (2.1)		1 (1.0)
*Corynebacterium spp*	1 (1.4)			1 (2.0)	1 (1.0)
*Staphylococcus pasteuri*		1 (4.0)		1 (2.0)	1 (1.0)
*Citrobacter freundii*	1 (1.4)			1 (2.0)	1 (1.0)
**Strict anaerobes**					
*Anaerobic bacilli*	9 (12.3)	3 (12.0)	9 (18.8)	3 (6.3)	12 (12.2)
*Anaerobic cocci*	1 (1.4)	2 (8.0)	3 (6.3)		3 (3.1)
*Anaerobic Streptococci*	1 (1.4)			1 (2.0)	1 (1.0)
**Aerobes**					
*Mycobacterium species*	1 (1.4)			1 (2.0)	1 (1.0)
**Total**	**73 (74.5)**	**25 (25.5)**	**48 (49.0)**	**50 (51.0)**	**98 (100.0)**

HIV + ve – HIV sero-positive, HIV-ve – HIV sero-positive.

**Table 3:** **The frequency distribution of bacterial isolates from HIV infected patients according to CD4+ cell count (n = 15)**

**Isolates**	**CD4+ cell counts**			
	**<200/mm** ^**3**^	**200 - 349/mm** ^**3**^	**350 - 499/mm** ^**3**^	**≥500/mm** ^**3**^
Viridans Streptococci group	0	0	1	4
*Streptococcus pneumoniae*	1	0	1	0
*Enterococcus species*	0	1	0	0
*Staphylococcus aureus*	0	0	2	1
*Anaerobic cocci*	0	0	0	2
*Proteus mirabilis*	0	1	0	0
*Escherichia coli*	0	0	0	1
*Staphylococcus epidermidis*	0	0	1	1
*Staphylococcus pasteuri*	1	0	0	0
*Anaerobic Bacilli*	1	0	0	2
**Total**	3	2	5	11

### Antibiotic use and susceptibility pattern

Most patients (n = 102, 78.5%) were on antibiotic therapy prior to recruitment into the study with about half (n = 49, 48%) possessing drug prescriptions (Table 1). The most commonly prescribed antibiotics were: metronidazole (n = 36), ampicillin/cloxacillin (n = 17) and ceftriaxone (n = 15). Susceptibility tests with 21 antibiotics were done on 68 (82.9%) aerobic/facultative anaerobic isolates (Table 4). Overall, the gram positive and gram negative isolates had poor susceptibility to cotrimoxazole and ampicillin, but high susceptibility to chloramphenicol (Table 4). All Viridans Streptococci isolates were resistant to penicillin G, sulfamethoxazole/trimethoprim (cotrimoxazole), ampicillin and tetracycline, but retained susceptibility to vancomycin. All *Staphylococcus aureus* strains were resistant to cotrimoxazole and, but susceptible to vancomycin, cefotaxime, linezolid, moxifloxacin and amoxicillin/clavulanate (Table 4). All the gram negative isolates (namely, *Escherichia coli*, *Klebsiella pneumonia*, *Proteus mirabilis* and *Citrobacter freundii*) were susceptible to amikacin and imipenem, but had poor susceptibility rates to ceftazidime, cotrimoxazole and ampicillin (Table 4).

**Table 4:** **The frequency distribution of bacterial isolates based on their susceptibility to different antibiotics (n = 68)**

	**Percentage susceptibilities of bacterial isolates**												
	**Gram negative isolates**					**Gram positive isolates**							
**Antibiotic**	**HI**	**PM**	**EC**	**CF**	**KP**	**ES**	**StA**	**StP**	**Stpy**	**VSG**	**SE**	**SA**	**SP**
Penicillin G	-	-	-	-	-	-	100	0.0	100	0.0	0.0	23.1	0.0
Amoxicillin/Clav.	100	50.0	0.0	-	0.0	-	-	-	-	-	50.0	100	0.0
Oxacillin						0.0	-	-	-	0.0	50.0	88.2	0.0
Ampicillin	0.0	50.0	0.0	0.0	0.0	66.7				0.0	0.0	0.0	0.0
Erythromycin	-	-	-	-	-	0.0	100	100	100	75.0	75.0	88.2	-
Clindamycin	-	-	-	-	-	0.0	-	100	-	92.9	50.0	50.0	100
Vancomycin	-	-	-	-	-	100	-	-	-	100	100	100	100
Gentamicin	0.0	100	60.0	0.0	0.0	33.3	-	-	-	66.7	75.0	93.8	0.0
Cotrimoxazole	0.0	50.0	0.0	0.0	0.0	0.0	0.0	0.0	0.0	0.0	25.0	0.0	0.0
Ciprofloxacin	83.3	100	70.0	0.0	25.0	0.0	-	100	-	84.6	75.0	88.2	0.0
Tetracycline	40.0	0.0	-	-	-	33.3	-	-	-	0.0	50.0	29.4	100
Ceftriaxone	60.0	100	30.0	-	0.0	-	-	100	-	81.8	-	-	-
Chloramphenicol	80.0	50.0	30.0	-	50.0	100	100	100	100	92.3	100	85.7	-
Levofloxacin	-	100	0.0	100	100	-	-	-	-	-	-	-	-
Amikacin	-	100	100	100	100	-	-	-	-	-	-	-	-
Imipenem	-	100	100	100	100	-	-	-	-	-	-	-	-
Cefuroxime	-	100	12.5	0.0	0.0	-	-	-	-	-	-	-	-
Ceftazidime	-	50.0	20.0	0.0	0.0	-	-	-	-	-	-	-	-
Cefotaxime	-	100	0.0	-	0.0	0.0	-	-	-	-	50.0	100	0.0
Linezolid	-	-	-	-	-	50.0	-	-	-	-	100	100	100
Moxifloxacin	-	-	-	-	-	50.0	-	-	-	-	100	100	-

Note. VSG – Viridans Streptococci Group, SA – Staphylococcus aureus, HI – Hemophillus influenza, EC – Escherichia coli, KP – Klebsiella pneumonia, SE – Staphylococcus epidermidis, SP – Staphylococcus pasteuri, ES – Enterococcus species, PM – Proteus mirabilis, StA – Streptococcus agalactiae, StP – Streptococcus pneumoniae, Stpy – Streptococcus pyogenes, CF – Citrobacter freundii.

### Fascial space involvement

The buccal space was the most frequently (32.4%) involved in the single space infections while the submasseteric space was more frequent (27%) in multiple space infections. Neither the HIV sero-status nor the CD4+ T lymphocyte cell count significantly influenced the frequency of fascial spaces involved with pyogenic odontogenic infection (p >0.05).

### Predictors of complications associated with pyogenic odontogenic infection

Twenty four (18.5%) of the patients had complications of respiratory distress due to the infections (Table 1); including 5 (3/2:female/male) who succumbed to death. The HIV infected patients were not significantly at risk of developing complications due to infections (OR, 0.68, 95% CI: 0.26 - 1.78, p = 0.43; Table 5). In bivariate analysis, age, trismus, multiple space infection, hospital admission and presence of fever were statistically significantly associated with complications of infection (p <0.05; Table 5). Multivariate logistic regression showed that impending airway obstruction (OR, 24.92, 95% CI: 3.80–163.40, p = 0.001) and admission status (OR, 20.76, 95% CI: 3.75–114.86, p = 0.001) were significant predictors of complications of infection.

**Table 5:** **The frequency distribution of patients according to risk factors associated with complications of pyogenic odontogenic infection (n = 130)**

**Variable**	**Category**	**Complications**		**OR (95% CI)**	
		**Yes**	**No**		**p value**
Age groups	≤35 years	11	84	4.5 (1.8-11.4)	
	>35 years	13	22		0.001
Sex	Female	11	51	1.10 (0.5-2.7)	
	Male	13	55		0.840
Trismus	Yes	20	52	5.2 (1.7-16.2)	
	No	4	54		0.002
Spaces^a^	Multiple	17	40	5.4 (1.8-15.7)	
	Single	5	63		0.001
Respiratory distress	Yes	17	5	49.1 (14.0 – 172.5)	
	No	7	101		<0.001
Hospital admission	Yes	17	7	34.3 (10.7-110.4)	
	No	7	99		<0.001
Bacterial culture	Positive	14	65	1.1 (0.5-2.8)	
	Negative	10	41		0.787
Nature of infection^b^	Polymicrobial	3	5	0.3 (0.1-1.5)	
	Monomicrobial	11	60		0.145
Fever on presentation	Yes	20	42	7.6 (2.4-23.9)	
	No	4	64		<0.001
Origin of the infection^c^	Mandible	20	65	3.1 (0.9-11.2)	
	Maxilla	3	30		0.076
HIV sero status	Positive	8	27	0.7 (0.3-1.8)	
	Negative	16	79		0.433

^a^n = 125, ^b^n = 79, n^c^ = 118.

## Discussion

### Demographic factors

In the present study, the male to female ratio was 1.1:1, which is similar to those of previous studies [26-28]. Most patients had primary level or no formal education and were from the low socioeconomic class (Table 1), which corroborates previous studies in Turkey [29] and India [30]. Patients of low socioeconomic status may be predisposed to poor nutrition with resultant impairment of host defense mechanism and may not afford preventive dental care. In the present study, the mean age of the patients was 29.5 years which is lower than values recorded in other studies [25,27]. Most patients (73.1%) were younger than 35 years, similar to what Pourdanesh et al. [28] observed in Iran.

### Predisposing factors for pyogenic odontogenic infection

All patients had pain and swelling on presentation, which corroborates previous studies [2,31]. Bridgeman et al. [32] postulated that a sudden onset of swelling in majority of the patients was the actual trigger for seeking health care. In the present study, trismus was recorded in 55.4% of patients and is frequently associated with masticatory and lateral pharyngeal space involvement [7]. In a comparable study in North India, Mathew et al. [33] revealed that trismus in odontogenic infection was frequently associated with upper airway obstruction, warranting critical patient appraisal and indeed in the present study, 27.8% of the patients presenting with trismus had respiratory distress.

In the present study, caries and post tooth extraction infections were the most common predisposing factors of pyogenic odontogenic infection (Table 1), which corroborates findings in a previous study [27]. The lower right and left third molars were the most frequently involved with caries, which supports earlier studies [27,28]. In our opinion, inadequate cleaning of the third molar teeth due to poor accessibility with cleaning devices may contribute to caries development. Similar to previous reports [19], we did not find any influence of HIV infection on post tooth extraction predisposing to pyogenic odontogenic infection.

### Bacterial isolates

In the present study 39.2% of the samples yielded no bacterial growth, a value much lower than 83.6% previously recorded in North India [33]. Negative cultures may be attributed to previous antibiotic use, improper specimen collection, wrong culture techniques and the presence of non-cultivable bacteria [5]. We recorded an average cultivable bacterial rate of 1.2 isolates per sample, similar to what Poeschl et al. [34] reported in Austria, but lower than 7.5 isolates observed in Thailand [35]. Eight of our cultures were polymicrobial (Table 1), with a predominance of facultative anaerobes (Table 2), which is in support of Kohli et al. [3].

Overall, the Viridans Streptococci Group and *Staphylococcus aureus* (Table 2) were the predominant isolates, which is in agreement with previous studies [21,30,34]. In corroboration to previous report in Austria [34], three quarters of the anaerobic isolates were bacilli. Although all *Proteus mirabilis* isolates were recovered from females, further studies are required to ascertain any gender difference in similar study samples. We, however, found no obvious influence of CD4+ T lymphocyte cell count on bacterial isolates in HIV infected patients.

### Antibiotic use and susceptibility pattern

In the present study, about three quarters of the patients had history of antibiotic use in pyogenic odontogenic infection prior to recruitment (Table 1) similar to previous studies in Spain [27], Iran [28], Italy [31] and West China [36]. Overall, more than half of the patients and approximately a third of HIV infected patients with history of prior antibiotic use did not possess prescriptions (Table 1) suggestive of self medication among the study population. This finding was similar to what Zhang et al. [36] reported in a prospective study in West China. The most frequently prescribed combinations in the descending order were ampicillin/cloxacillin/metronidazole, ceftriaxone/metronidazole, amoxicillin/metronidazole and gentamicin/metronidazole, which corroborates a study [37] in Bulgaria, where a beta lactam and metronidazole was the most prescribed empirical combination. A previous survey on prescription pattern among Ugandan dental health professionals [38] had similar findings. However, in a Spanish study, Sanchez et al. [27] found majority of patients using amoxicillin–clavulanate combination prior to admission.

In the present study, all Viridans Streptococci isolates demonstrated resistance to penicillin G, cotrimoxazole and ampicillin (Table 4), contrary to a previous report [8]. On the other hand, all *Staphylococcus aureus* strains exhibited susceptibility to vancomycin, cefotaxime, linezolid, moxifloxacin and amoxicillin/clavulanate, but retained resistance to ampicillin and cotrimoxazole (Table 4), similar to a previous study [8]. Reasons for bacterial resistance to antibiotics include: overuse of antibiotics in humans, unnecessary prescriptions, the presence of antibiotics in food and water supplies, and mutation and/or exchange of genes within the genus [39]. All *Streptococcus pneumoniae* isolates showed susceptibility to chloramphenicol, clindamycin, ceftriaxone, erythromycin and ciprofloxacin, while being resistant to penicillin G and cotrimoxazole (Table 4). In a previous study [40] in Malawi, *Streptococcus pneumonia* isolates exhibited lower values of resistance to cotrimoxazole. *Escherichia coli* isolates were highly susceptible to imipenem, amikacin, ciprofloxacin and gentamicin, but all exhibited resistance to amoxicillin/clavulanate, ampicillin, cotrimoxazole, cefotaxime and levofloxacin (Table 4). Similarly, a previous report [41] in Ethiopia showed *Escherichia coli* isolates to have high resistance to cotrimoxazole as well as erythromycin, amoxicillin and tetracycline. Gagliotti et al. [42] asserted that this trend of resistance may be explained by the spread of multidrug resistant plasmids containing genes for the Extended spectrum beta lactamase production. Generally, most bacterial isolates were resistant to cotrimoxazole (Table 4), which could be due to the frequent prescription of the drug especially among the Ugandan dental health practitioners [38] and the prophylaxis against *Pneumocystis jiroveci* pneumonia in HIV infected patients [15,43].

### Fascial spaces

In the present study, the three most involved spaces in descending order of frequency were the buccal, submasseteric and the submandibular spaces (Table 6). Previous studies reported the submandibular [21,33] and buccal spaces [1,3,28] to be the most frequently affected. In the present study, buccal space was significantly associated with the out-patients while submandibular, sublingual, parapharyngeal, retropharyngeal and multi space infections were significantly associated with the in-patient group, similar to a report in New Zealand [44].

**Table 6:** **The frequency distribution of patients according to infected fascial spaces, HIV sero-status, sex and hospital admission status (n = 130)**

**Fascial space**	**HIV + ve n (%)**	**HIV-ve n (%)**	**Female n (%)**	**Male n (%)**	**Outpatient n (%)**	**Inpatient n (%)**
Buccal	14 (6.8)	38 (18.5)	28 (13.7)	24 (11.7)	47 (22.9)	5 (2.4)
Submasseteric	9 (4.4)	37 (18.1)	14 (6.8)	32 (15.6)	38 (18.5)	8 (3.9)
Submandibular	14 (6.8)	22 (10.7)	18 (8.9)	18 (8.9)	21 (10.2)	15 (7.3)
Temporal	4 (2.0)	11 (5.4)	6 (2.9)	9 (4.4)	10 (4.9)	5 (2.4)
Canine	4 (2.0)	9 (4.4)	5 (2.4)	8 (3.9)	12 (5.9)	1 (0.5)
Parapharyngeal	5 (2.4)	7 (3.4)	5 (2.4)	7 (3.4)	3 (1.5)	9 (4.4)
Pretracheal	2 (1.0)	4 (2.0)	4 (2.0)	2 (1.0)	0 (0.0)	6 (2.9)
Peritonsillar	0 (0.0)	5 (2.4)	3 (1.5)	2 (1.0)	4 (2.0)	1 (0.5)
Pterygoid	1 (0.5)	4 (2.0)	2 (1.0)	3 (1.5)	3 (1.5)	2 (1.0)
Parotid	1 (0.5)	3 (1.5)	4 (2.0)	0 (0.0)	3 (1.5)	1 (0.5)
Submental	0 (0.0)	3 (1.5)	2 (1.0)	1 (0.5)	3 (1.5)	0 (0.0)
Sublingual	1 (0.5)	1 (0.5)	0 (0.0)	2 (1.0)	0 (0.0)	2 (1.0)
Retropharyngeal	1 (0.5)	1 (0.5)	1 (0.5)	1 (0.5)	0 (0.0)	2 (1.0)
Labial	2 (1.0)	0 (0.0)	2 (1.0)	0 (0.0)	2 (1.0)	0 (0.0)
Periorbital	0 (0.0)	2 (1.0)	0 (0.0)	2 (1.0)	2 (1.0)	0 (0.0)
Single	18 (14.4)	50 (40.0)	39 (31.2)	29 (23.2)	61 (48.8)	7 (5.6)
Multiple	16 (12.8)	41 (32.8)	22 (17.6)	35 (28.0)	40 (32.0)	17 (13.6)

HIV + ve – HIV sero-positive, HIV-ve – HIV sero-positive.

Although in the present study, the HIVsero-status did not influence specific fascial space involvement, Srivanitchapoom et al. [25] revealed that the submandibular space was the most frequently involved in non HIV infected patients, whereas the superficial masticator space was the most frequently affected in HIV infected counterparts. They further observed that immuno-compromised patients tended to develop multiple space infections more often than normal counterparts contrary to our findings. Additionally, we observed a statistically significant association between the outcome of death and retropharyngeal space involvement probably due to the spread of infection into the mediastinum [45] with respiratory distress. The predominance of buccal space infections in the present study may be attributed to the large number of out-patients compared to inpatients as opposed to previous studies because buccal space infections have less risk of compromising the airway which is the common reason of patient admission for odontogenic infection [45].

### Predictors of complications of pyogenic odontogenic infection

In the present study, patients with impending airway obstruction on admission were likely to develop complications such as respiratory distress, a similar finding reported in Taiwan [46]. Multiple fascial space involvement has been noted to be significantly associated with life threatening complications [33]. However, in the present study there was no significant association between multiple space infections and complications of infection, probably due to other uncontrolled confounding effects. We recorded a mortality rate of 3.8% (n = 5), which is at variance with previous studies : 11.25% [47] and 7.7% [48] in Brazil; 1.6% [1] and 1% [46], Taiwan; 0.27%, Italy [26] and 0.9%, India [21].

## Conclusions

In the present study, dental caries was the most common predisposing factor for pyogenic odontogenic infection, which requires community oral health education. Overall, there was high bacterial resistance to tetracycline, penicillin G, ceftazidime, ampicillin and cotrimoxazole, suggesting the need for susceptibility tests of isolates and rational use of antibiotic in the management of these infections. Prevention of the infections requires strengthening of oral health in the community.
